# Laser Sensing and Chemometric Analysis for Rapid Detection of Oregano Fraud

**DOI:** 10.3390/s23156800

**Published:** 2023-07-30

**Authors:** Luca Fiorani, Antonia Lai, Adriana Puiu, Florinda Artuso, Claudio Ciceroni, Isabella Giardina, Fabio Pollastrone

**Affiliations:** Diagnostics and Metrology Laboratory, Physical Technologies for Safety and Health Division, Fusion and Technology for Nuclear Safety and Security Department, ENEA, Via Enrico Fermi 45, 00044 Frascati, Italy; antonia.lai@enea.it (A.L.); adriana.puiu@enea.it (A.P.); florinda.artuso@enea.it (F.A.); claudio.ciceroni@enea.it (C.C.); isabella.giardina@enea.it (I.G.); fabio.pollastrone@enea.it (F.P.)

**Keywords:** quantum cascade laser application, laser spectroscopy, photoacoustic technique, multivariate calibration, agrofood chain, food fraud

## Abstract

World health is increasingly threatened by the growing number of spice-related food hazards. Further development of reliable methods for rapid, non-targeted identification of counterfeit ingredients within the supply chain is needed. ENEA has developed a portable, user-friendly photoacoustic laser system for food fraud detection, based on a quantum cascade laser and multivariate calibration. Following a study on the authenticity of saffron, the instrument was challenged with a more elusive adulterant, olive leaves in oregano. The results show that the reported method of laser sensing and chemometric analysis was able to detect adulterants at mass ratios of at least 20% in less than five minutes.

## 1. Introduction

The global spice market is a significant and diverse industry that plays a vital role in the global economy [1]. Spices have been valued for centuries for their flavour-enhancing properties and their use in culinary traditions around the world. The global spice market has experienced steady growth over the years. Demand for spices is driven by changing consumer preferences, the expansion of international cuisines and growing awareness of the health benefits associated with spices. The market size is influenced by factors such as population growth, disposable income and urbanisation [2]. The spice market faces challenges such as climate change affecting crop yields, pest and disease management, and supply chain disruptions. In addition, ensuring fair trade practices, addressing food safety concerns and maintaining quality standards are ongoing challenges for the industry.

Oregano is one of the spices that has been found to be frequently adulterated [3]. Adulteration of oregano usually involves the addition of cheaper substances or fillers, which can dilute the quality or change the composition of the spice. These fillers may include substances such as olive leaves, myrtle leaves, sumac leaves or other less expensive herbs. These fillers are mixed with real oregano to increase the overall weight of the product. Many strategies have been used to detect food fraud [4], but non-targeted approaches using fast spectral techniques combined with chemometric analysis tools should be used to develop identification tests that work in industrial settings: rapid confirmatory methods can provide verification of critical anomalies before an ingredient is removed from the supply chain [5].

In every measurement process, the question arises whether to prioritise data quality or reduce measurement time. In the first case, preference should be given to classical analytical techniques, generally carried out in laboratories far away from the sampling areas. In the second case, rapid optical instruments can be used in the field, taking into account their limitations, e.g., possible low technological maturity and relatively high detection limits.

In this context, the Diagnostics and Metrology Laboratory (FSN-TECFIS-DIM) of the Italian National Agency for New Technologies, Energy and Sustainable Economic Development (ENEA) [6] is using laser photoacoustic spectroscopy (LPAS) [7] for the rapid detection of food fraud [8]. Typically, in an LPAS setup, a laser beam is modulated at an audio frequency and injected into a resonant cell, where it strikes the sample under investigation, which absorbs the incident radiation. As a result, the illuminated area experiences an increase in temperature and volume, creating a pressure wave that is detected by a microphone connected to a lock-in amplifier synchronised with the modulator. The output signal is proportional to the absorption of the sample. The spectrum is usually obtained in the “fingerprint region”–a broad band of the infrared (IR) where many organic compounds can be detected–simply by changing the laser wavelength. LPAS differs from conventional IR spectroscopy by the key advantage of unrivalled source power (laser vs. lamp). Recently, a QCL (quantum cascade laser)-based LPAS system has been developed at ENEA [9], which has the following characteristics: rapidity, sensitivity, specificity, simplicity, repeatability, in situ measurement, uncomplicated sampling, ease of use and cost-effectiveness. The system has been used to detect saffron fraud [10] and to identify the production areas of different olive cultivars [11].

Literature on the detection of adulterated oregano is less abundant than that for saffron; however, the main analytical techniques have been tried. Adulterations were searched with Fourier-transform infrared spectroscopy (FT-IR) and liquid chromatography-high resolution mass spectrometry LC-HRMS [12], ambient mass spectrometry (AMS) and direct analysis in real time-high resolution mass spectrometry (DART-HRMS) [13], near-infrared spectroscopy (NIR) and mid-infrared spectroscopy (MIR), hyperspectral imaging (HSI), gas chromatography coupled to mass spectrometry (GC-MS) and proton-transfer-reaction time-of-flight mass spectrometry (PTR-TOF-MS) [14] and next generation sequencing (NGS) and nuclear magnetic resonance (NMR) [15].

Unfortunately, all these methods require sample preparation and destruction, must be carried out by trained personnel (research scientists, laboratory technicians, police officers, etc.), are typically complex, expensive and time-consuming, and cannot be used in the field.

In particular, the most widely used existing techniques fall into the two families of chromatography (gas or liquid) [16] and mass spectrometry [17], both of which have the following disadvantages:

- Cost: chromatography and mass spectrometry can be expensive to set up and maintain. Chromatography equipment, consumables, and solvents can add up to significant costs, especially for high-performance systems. Purchasing a mass spectrometer and ensuring its proper functioning may pose financial challenges for smaller research facilities or laboratories.
- Time-consuming: chromatographic separations and mass spectrometry sample preparation can be time-consuming, especially for complex mixtures. The process may take hours or even days to complete, depending on the complexity of the sample, which can introduce the potential for mistakes or loss of analytes during the process.
- Complexity: both chromatography and mass spectrometry are complex techniques that require skilled operators and a deep understanding of the principles behind the methods. Data analysis can be complicated, and it often requires specialised software and expert personnel to handle the measurements effectively.
- Sample preparation: both chromatography and mass spectrometry often require careful sample preparation to ensure accurate results. Preparing samples can be labour-intensive and may introduce errors if not conducted correctly.
- Sensitivity: both chromatography and mass spectrometry might lack the sensitivity needed to detect low-abundance compounds in a mixture. This limitation can be an issue when analysing trace amounts or minor components in a sample.

Chromatography has also been criticised for resolution limitations, potential for sample degradation, lack of reproducibility and hazardous waste generation. Other issues raised about mass spectrometry include possible interferences, large differences in ionisation efficiency from one molecule to another, fragmentation of ions and high vacuum requirements.

The motivation for this work is precisely to provide an alternative to existing techniques that would allow fraud of a certain magnitude to be revealed by unskilled personnel in production, transit, or sales areas within minutes. Ideally, industry stakeholders (agrofood enterprises, inspection authorities, customs agencies…) could use a portable LPAS system for initial field screening of food authenticity.

The detection limit of the existing techniques is in the order of 10%, i.e., it is difficult to detect an adulterant of less than 10% of the total mass, while the detection limit of the LPAS system is less than 20%, as will be shown. All in all, the price to be paid in terms of sensitivity for having a technique with the important advantages of speed, ease of use and deployability in the field is not that high, partly because, unlike saffron, economically motivated adulteration (EMA) of oregano is only practical at high levels.

## 2. Materials and Methods

### 2.1. LPAS System

**Figure 1:**
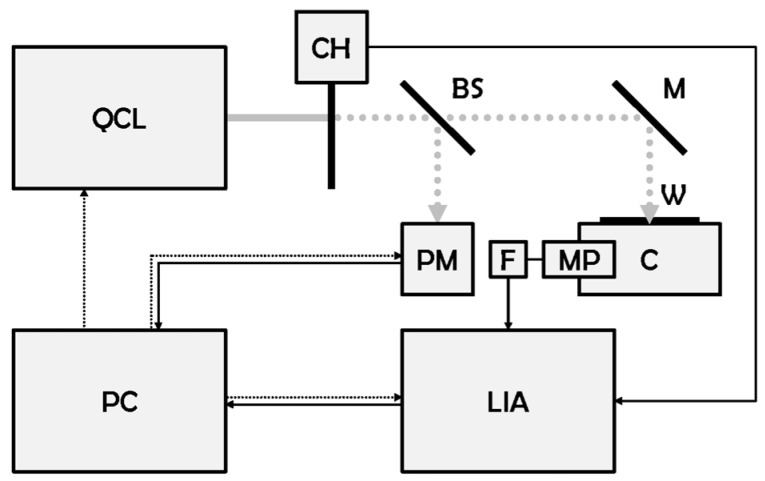
Block diagram of the LPAS system (old and new). BS: beam splitter, C: photoacoustic cell, CH: chopper, F: active low pass filter, LIA: lock-in amplifier, M: mirror, MP: microphone, PC: personal computer, PM: power meter, QCL: quantum cascade laser, W: window. Grey continuous line: continuous wave laser beam, grey dotted line: modulated laser beam, black continuous line: signal, black dotted line: control.

**Figure 2:**
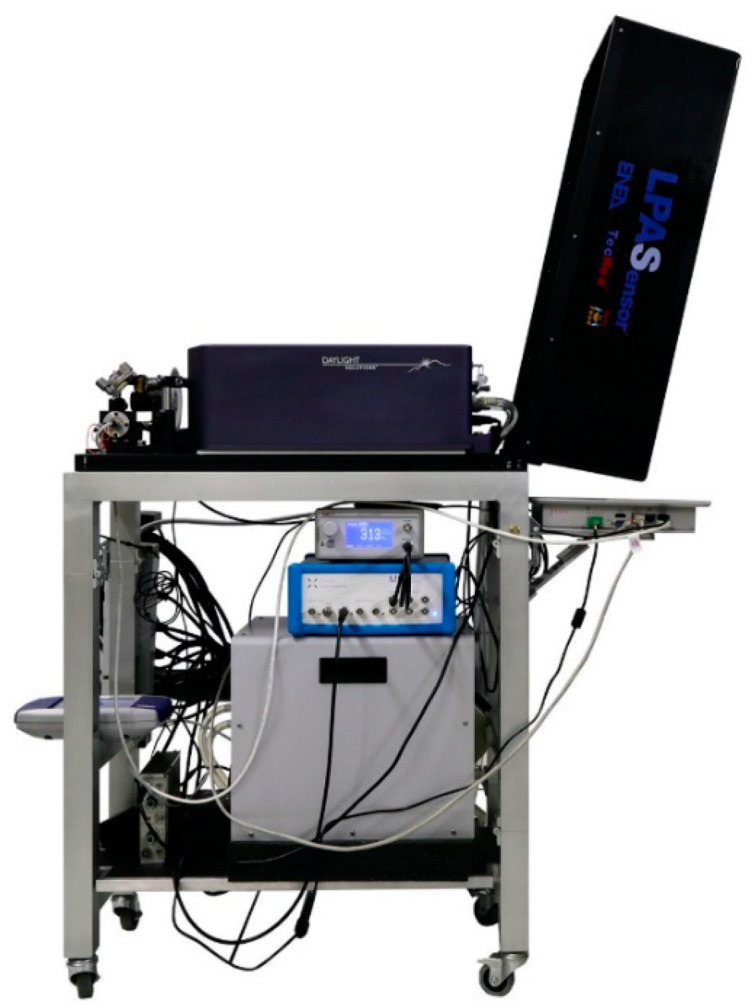
New LPAS system mounted on a trolley. The legs are foldable. Supports and subsystems can be quickly disassembled and placed in two fly cases for transport.

The first LPAS sensor, called the “old system”, has been described in this journal [10]: only its block diagram is recalled in Figure 1. A second LPAS sensor, called the “new system”, was later developed. The second has the same architecture as the first, but with many improvements:

- It is mounted on a trolley and can therefore be used in the field (Figure 2);
- The cell has been redesigned to facilitate sample loading using a small drawer;
- Almost all subsystems have been replaced by new models with improved performance (Table 1);
- The wavelength range of the laser source has been extended (Table 2).

The diameter and height of the cell, made of black anodised aluminium, are 17.1 and 13.0 mm, respectively. Microphone sensitivity is better than −55 dB between 0.3 and 7 kHz (relative to 1.0 V/0.1 Pa). Its maximum of −48 dB is at 4.5 kHz. The lock-in amplifier was set with the following parameters: a centre frequency of 313 Hz, with external reference from the chopper, and a 3-order filter with a bandwidth of 1 Hz.

As usual, the LPAS system was tested with a standard material (activated carbon), before being challenged with the simulation of the most common fraud of the spice under investigation: dry oregano leaflets adulterated with chopped dry olive leaves. Three measurement runs were carried out between July 2020 and June 2021.

### 2.2. Measurement Run of July 2020 (“07-2020”)

The rationale behind this first measurement run was to calibrate the sensor and examine commercial samples. Bearing in mind that oregano fraud is not economically viable at low levels of adulteration, it was decided to retain the 20% limit in subsequent samples prepared as follows:

1. 0% (OR): Oregano leaflets with ICEA (https://icea.bio/en/ accessed on 4 July 2023) certificate of conformity were purchased from Bioagricola Bosco (Favara AG, Italy) and ground using a ball pestle impact grinder;
2. 100% (OL): Ground olive leaves were purchased from Sigma–Aldrich (olive leaf dry extract 1478265);
3. 20%, 60%, 80%, 90% and 95%: Mixtures with OL/(OR + OL) mass ratios of 20%, 60%, 80%, 90% and 95%, respectively, were prepared using a high-precision analytical balance;
4. DA, DJ and GR: commercial ground oregano sold by three different companies.

Samples 1 to 3 were used to calibrate the sensor for measuring the concentration of olive leaves in oregano. As explained in Section 3.1, the calibration was performed with a linear fit.

### 2.3. Measurement Run of February 2021 (“02-2021”)

Although the results of 07-2020 were encouraging (see Section 3.1), their samples were questionable as they represented only one olive cultivar and one oregano variety.

To overcome the first shortcoming, four olive cultivars from three different fields under ENEA control (“Mario”, “Rolando”, and “ENEA”) and three oregano varieties were procured:

- Olive
  ○ OL-CM: Canino cultivar from the Mario field;
  ○ OL-CR: Canino cultivar from the Rolando field;
  ○ OL-FM: Frantoio cultivar from the Mario field;
  ○ OL-FR: Frantoio cultivar from the Rolando field;
  ○ OL-LE: Leccino cultivar from the ENEA field;
  ○ OL-LM: Leccino cultivar from the Mario field;
  ○ OL-LR: Leccino cultivar from the Rolando field;
  ○ OL-ME: Maurino cultivar from the ENEA field;
  ○ OL-MM: Maurino cultivar from the Mario field;
  ○ OL-MR: Maurino cultivar from the Rolando field.
- Oregano
  ○ OR-CA: oregano twigs (impossible to adulterate) from Cosenza CS, Italy;
  ○ OR-FA: oregano twigs from Favignana TP, Italy;
  ○ OR-ST: oregano sample of 07-2020 from Favara AG, Italy.

Both olive leaves and oregano leaflets (detached from the twigs, if necessary) were ground with a ball pestle impact grinder. In this way, Italian oregano (coming mainly from southern Italy) and central Italian olive were better represented. After a consistency check (see Section 3.2.1), new samples were prepared as follows:

1. OL: obtained by mixing cultivars in these proportions to reproduce an average composition of central Italy: Frantoio 40%, Leccino 30%, Maurino 20% and Canino 10%;
2. OR: obtained by mixing varieties in these proportions to reproduce an average composition of southern Italy: Cosenza 34%, Favignana 33% and Favara 33%;
3. OL20inOR, OL60inOR, OL80inOR, OL90inOR and OL95inOR: blends with OL/(OL + OR) mass ratios of 20%, 60%, 80%, 90% and 95%, respectively, obtained using a high-precision analytical balance.

Samples 1 to 3 were used to calibrate the sensor for measuring the concentration of olive leaves in oregano. As explained in Section 3.2, the calibration was performed with chemometric analysis.

### 2.4. Measurement Run of June 2021 (“06-2021”)

The measurements were repeated in June 2021. The samples had small differences:

1. OL: obtained by mixing cultivars in these proportions to reproduce an average composition of central Italy: Pendolino 40%, Leccino 30%, Maurino 20% and Canino 10% (Pendolino, also coming from ENEA controlled fields, replaced Frantoio due to the lack of the latter cultivar);
2. OR: obtained only from the Cosenza variety (this variety was chosen to test the sensor because its spectrum is closer to that of olive, see Section 3.2.1);
3. OL20inOR, OL60inOR, OL80inOR, OL90inOR and OL95inOR: blends with OL/(OL + OR) mass ratios of 20%, 60%, 80%, 90% and 95%, respectively, obtained using a high-precision analytical balance.

Samples 1 to 3 were used to calibrate the sensor for measuring the concentration of olive leaves in oregano. As explained in Section 3.3, the calibration was performed with chemometric analysis.

## 3. Results 

The spectrum of each sample (LPAS signal vs. wavelength) was obtained in three steps:

1. The QCL scanned wavelengths from λ_1_ to λ_2_ with a step size of Δλ.
2. The lock-in amplifier and the power meter measured the photoacoustic signal (V) and the laser power (W), respectively, at each wavelength. Each measurement took 1 s and was repeated N times, and these measurements were averaged.
3. The LPAS signal (V/W) is given by the ratio of these averages (thus normalising the photoacoustic signal to the laser power).

### 3.1. Measurement Run 07-2020

The measurement parameters for this run are as follows (high-resolution spectra):

- λ_1_ = 7.525 μm;
- λ_2_ = 10.000 μm;
- Δλ = 0.025 μm;
- N = 10;
- One spectrum per sample was measured, and each spectrum was acquired in less than twenty minutes.

Although the LPAS system can emit between 6 and 11.1 μm, measurements were carried out between 7.5 and 10 μm for two reasons: (i) the QCL showed undesirable fluctuations between 6 and 7.5 μm and low power between 10 and 11.1 μm; (ii) the differences between the absorption spectra of oregano and olive leaves appeared minimal in both the 6–7.5 and 10–11.1 μm ranges.

**Figure 3:**
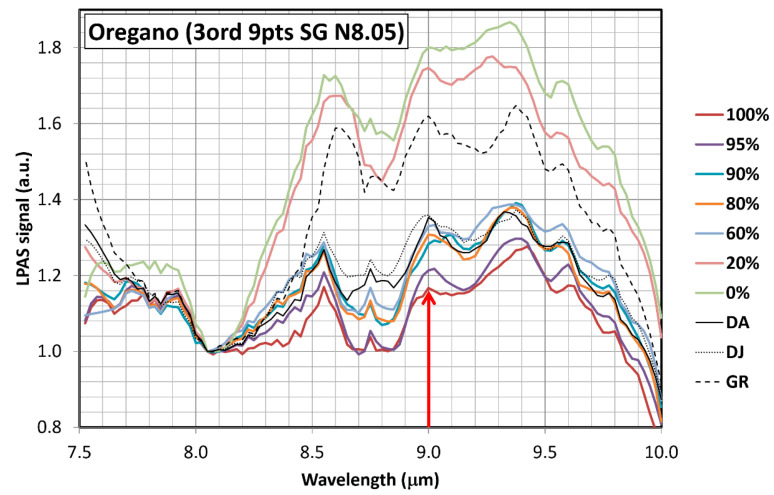
Spectra of oregano “0%” (OR), olive leaves “100%” (OL) and mixtures with OL/(OR + OL) mass ratios of 20%, 60%, 80%, 90% and 95%. Third-order Savitzky–Golay smoothing on 9 points and normalisation to 8.05 μm have been applied. The only spectral region where evidently the value of the LPAS signal decreases monotonically with olive leaves concentration is around 9.0 µm. At 9.6 µm, for example, starting from the highest to the lowest LPAS signal, the following concentrations of olive leaves are found: 0%, 20%, 60%, 90%, 80%, 95% and 100%, i.e., the absorption for the sample with 90% olive leaves is higher than expected.

**Figure 4:**
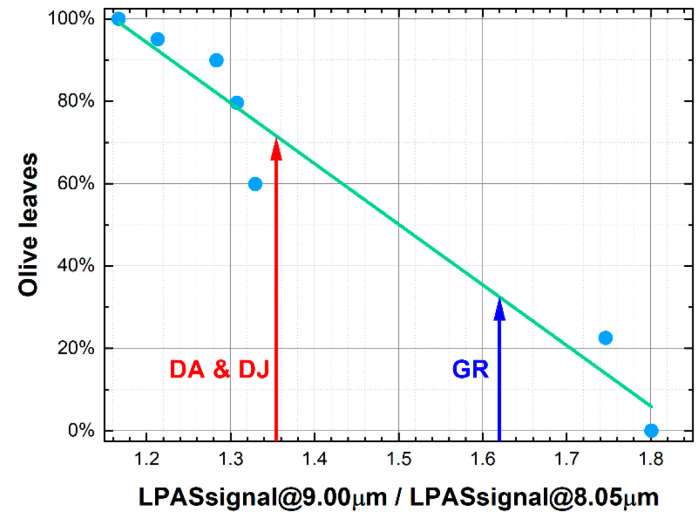
Olive leaves concentration vs. LPAS signal at 9 μm (normalised to 8.05 μm). The commercial samples do not seem pure. The green line is the linear fit having intercept: 2.7 ± 0.2; slope: −1.4 ± 0.1; R-squared: 0.954.

The spectra–after third-order Savitzky–Golay smoothing on 9 points and normalisation to 8.05 μm–are shown in Figure 3. Unfortunately, spectra are quite entangled. Moreover, the morphological differences between the curves of oregano and olive leaves are rather small: the peaks have more or less the same positions, but the intensity of absorption changes between about 8 and 10 μm. Nevertheless, at 9 μm, the value of the LPAS signal decreases monotonically with olive leaves concentration and a linear relationship was found between these two variables (Figure 4). Related to linear fitting, R-squared (also known as the coefficient of determination) is a statistical measure of how well the straight line matches the observed data [18]. Its maximum value is 1, and the higher it is, the better the fit. Residuals and distances of the dependent variable are first calculated to obtain it. A residual is the difference between the observed value and the predicted value, while a distance is the difference between the observed value and the mean of the observed values. Then, the residual sum of squares (RSS) and the total sum of squares (TSS) are computed: RSS is the sum of the residuals squared, and TSS is the sum of the distances squared. Finally, R-squared is 1–RSS/TSS.

The fit is quite encouraging, suggesting that the application of chemometric analysis could be successful. In addition, we can conclude with reasonable certainty that the commercial samples are fraudulent: slightly GR and heavily DA and DJ.

### 3.2. Measurement Run 02-2021

#### 3.2.1. Consistency Check

Prior to chemometric analysis, the consistency of the data was checked using high-resolution spectra. The measurement parameters for this run are as follows:

- λ_1_ = 7.525 μm;
- λ_2_ = 10.000 μm;
- Δλ = 0.025 μm;
- N = 5;
- Three spectra per sample were measured, and each spectrum was acquired in less than ten minutes.

**Figure 5:**
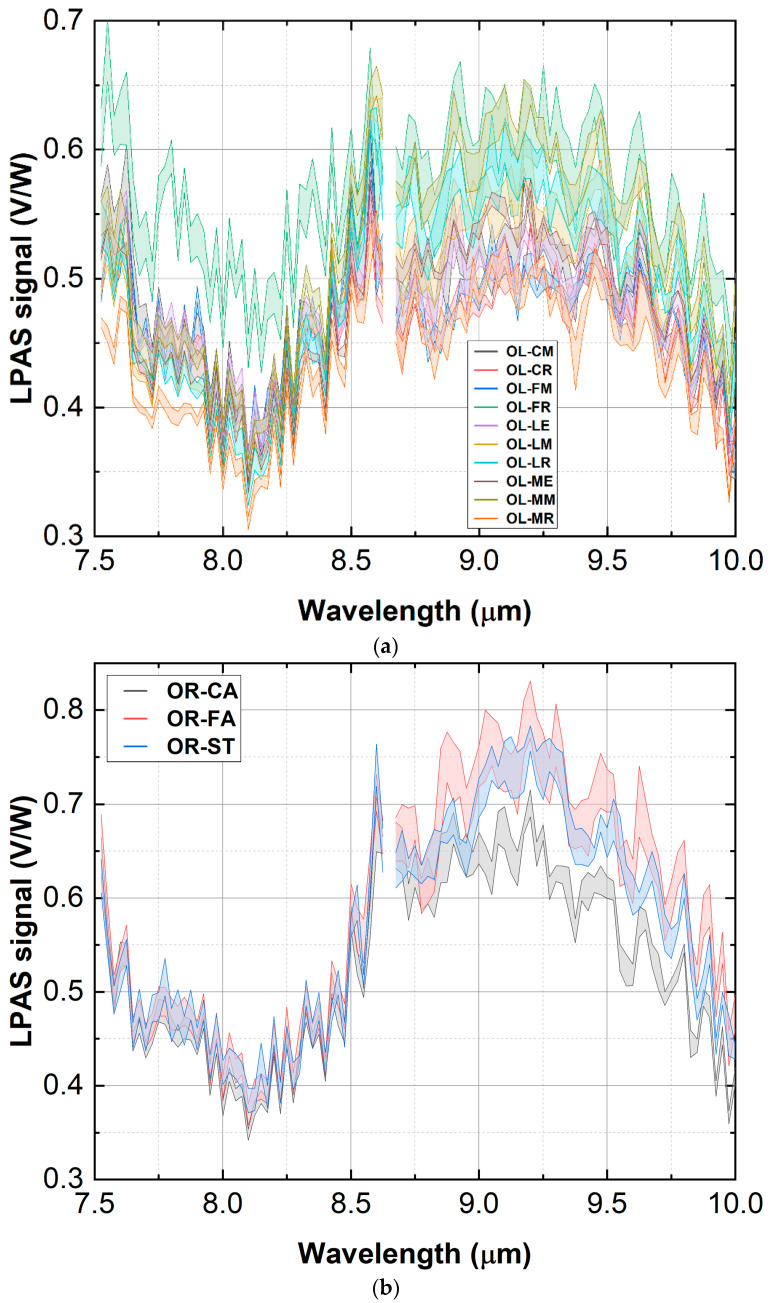

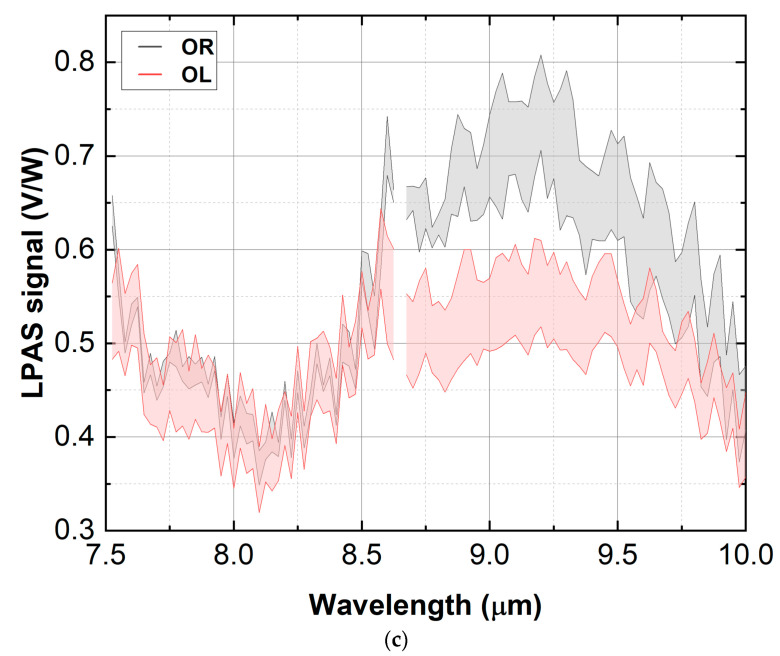
Spectra of olive cultivars (**a**), oregano varieties (**b**) and olive and oregano averages (**c**). Spectra are shown as bands centred on the mean value of the measurements and having a thickness equal to the standard deviation. The emission between 7.5 and 10 µm is carried out by two laser modules, and the switch that takes place around 8.65 µm results in a fluctuation of power: the corresponding points of the spectra have been eliminated to avoid artefacts.

The spectra are shown in Figure 5 as bands centred on the mean of the measurements and with a thickness equal to the standard deviation. Although some olive cultivars display a more intense LPAS signal, Figure 5a shows that the pattern of the curves is the same for all of them. Since the samples of the various olive cultivars were mixed in the subsequent investigation, the purpose of Figure 5a was simply to show that the spectra of the various cultivars are similar and, therefore, it is justified to mix them, which is why it was not considered a problem if they do not stand out totally in the graph. Slightly different is the case with oregano shown in Figure 5b: the OR-CA sample shows a somewhat less intense LPAS signal between 9 and 10 μm. However, the oregano spectra were considered to be sufficiently consistent: in the region around 9 μm, the average of the oregano varieties differs significantly from the average of the olive cultivars, as can be seen in Figure 5c. The same graph also shows that the spectra of olive and oregano are morphologically similar, i.e., they have the same general pattern despite a difference in intensity of around 9 µm. The situation is different when spice and adulterant have totally different peaks, as was the case with saffron mixed with turmeric and tartrazine (an artificial compound) [10].

#### 3.2.2. Chemometric Analysis

The chemometric analysis was performed using two classical methods: principal component analysis (PCA) and partial least squares regression (PLS), after standard normal variate (SNV) pretreatment [19]. The application of PLS was preceded by PCA to check if it was possible to identify principal components from the experimental data. PCA and PLS were run on the experimental data using OriginPro [20]. PLS results have been verified with ChemFlow [21]. Attempts to improve the PLS results by applying a Savitzky-Golay smoothing and differentiation filter of various orders and with different numbers of points [19] were unsuccessful.

Once it has been checked that the spectra do not show particularly narrow peaks, the chemometric analysis can be carried out with low-resolution measurements, thus saving substantial time (we recall that fast operation is one of the aims of the LPAS system). The measurement parameters for this run are as follows:

- λ_1_ = 7.6 μm;
- λ_2_ = 10.0 μm;
- Δλ = 0.1 μm;
- N = 10;
- Ten spectra per sample were measured, and each spectrum was acquired in less than five minutes).

**Figure 6:**
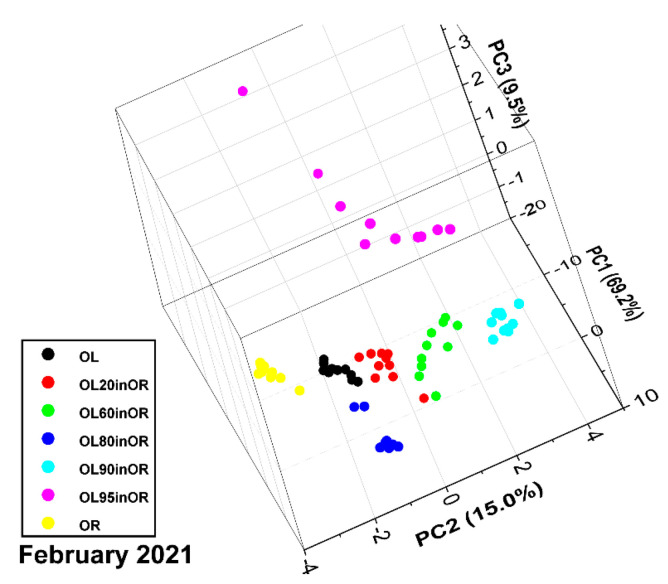
PCA score plot of the spectra of oregano, olive leaves and their mixtures (measurement run 02-2021).

The PCA score plot is shown in Figure 6. Probably due to nonlinear effects, as discussed in a similar study on saffron, the scores for the mixtures are not aligned with the segment that connects points for pure oregano and pure olive leaves [10]. However, apart from a few isolated points, the point clouds are reasonably separated. The first three principal components explain 93.7% of the variance. The PCA results give hope that PLS can correctly predict concentrations, remembering that, according to [19], “With multivariate calibration, more than one wavelength is used allowing correction of spectral interferences and other matrix effects, such as chemical interactions... Depending on the degree of nonlinearities, linear multivariate regression may be able to correct the nonlinear deviations”.

**Figure 7:**
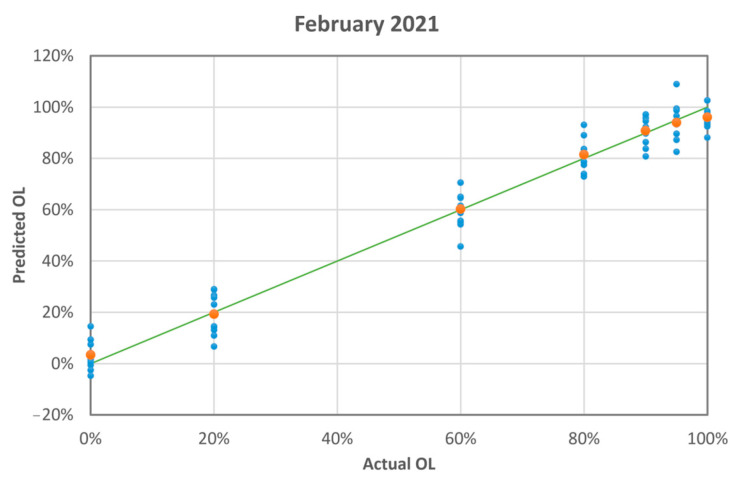
The blue dots are the PLS-predicted mass ratio of olive leaves vs. the actual mass ratio (measurement run 02-2021). The orange dots are the averages of the blue dots. The green line is the linear fit of the orange dots having intercept: 0.02 ± 0.02; slope: 0.97 ± 0.02; R-squared: 0.998.

PLS converged with eight factors, thus explaining 98.1% of the variance for x (effects) variables and 96.6% of the variance for y (responses) variables. Convergence was assessed by the root mean square of the predicted residual sum of squares (PRESS). Figure 7 shows the difference between the predicted and actual adulterant mass ratio. The maximum absolute difference is 14%. Table 3 summarises the PLS results. The absolute difference, i.e., the absolute value of the difference between the observed value and the predicted value (residual), is less than the standard deviation for all samples. The mean of absolute differences and standard deviations is 1.7% and 3.7%, respectively.

### 3.3. Measurement Run 06-2021

The measurement parameters for this run are as follows:

- λ_1_ = 7.5 μm;
- λ_2_ = 10.0 μm;
- Δλ = 0.1 μm;
- N = 3;
- Twenty-five spectra per sample were measured, and each spectrum was acquired in less than two minutes.

As can be seen, it was decided to obtain more spectra (25 instead of 10), and thus populate the PCA score plots with more points and try to resolve some ambiguities at the expense of measurement accuracy, which is now improved only with three repetitions instead of ten. In this way, however, the operation time for each sample is more or less the same (25 × 3 measurements instead of 10 × 10 measurements).

**Figure 8:**
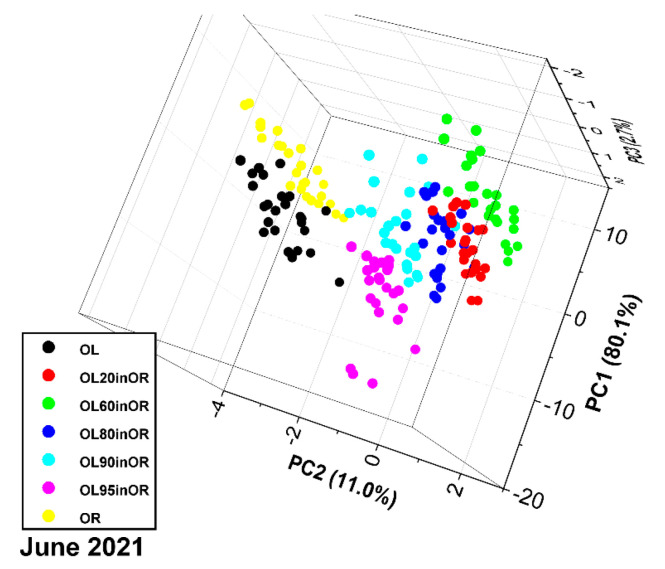
PCA score plot of the spectra of oregano, olive leaves and their mixtures (measurement run 06-2021).

The PCA score plot is shown in Figure 8. The first three principal components explain 93.8% of the variance. For run 06-2021, similar considerations apply as for run 02-2021, especially regarding nonlinearity. Furthermore, on the one hand, the point clouds have more overlap; on the other hand, the samples with 60%, 80%, 90% and 95% adulterants are arranged approximately along a straight line. The discrepancy between pure components and mixtures, already observed in the previous run, may be due to physicochemical phenomena related to mixing that alter the photoacoustic response of the sample.

**Figure 9:**
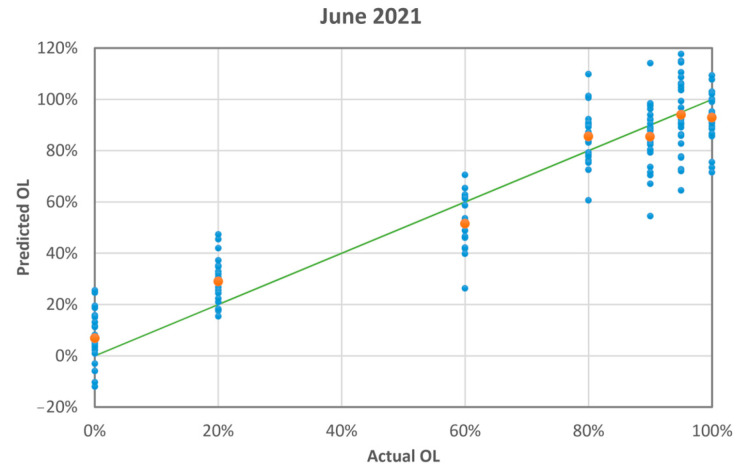
The blue dots are the PLS-predicted mass ratio of olive leaves vs. the actual mass ratio (measurement run 06-2021). The orange dots are the averages of the blue dots. The green line is the linear fit of the orange dots having intercept: 0.08 ± 0.04; slope: 0.88 ± 0.06; R-squared: 0.977.

PLS converged with six factors, thus explaining 96.1% of the variance for x (effects) variables and 87.9% of the variance for y (responses) variables. Figure 9 shows the difference between the predicted and actual adulterant mass ratio. The maximum absolute difference is 36%. Table 4 summarises the PLS results. The absolute difference is less than the standard deviation in five cases out of seven. The mean of absolute differences and standard deviations is 6.1% and 7.6%, respectively.

## 4. Discussion

The PLS prediction of the second run is worse. Intercept and slope deviate more from the expected value, i.e., 0 and 1, respectively, the errors of intercept and slope are larger, and R-squared is smaller. Going from ten to three measurements, one actually expects a worsening of the standard deviation equal to the square root of 10/3, i.e., 1.8. In reality, the mean standard deviation increases from 3.7 to 7.6, which equals a worsening of 2.1, greater than 1.8.

Although this can be partly explained by a more unfavourable variety of oregano, as discussed in Section 2.3, it is likely that the worse initial quality of the spectra propagated non-linearly along the calculation chain, leading to less satisfactory results.

The lesson learnt from these two measurement runs is that the LPAS system may not need many repeated measurements to detect fraud, potentially increasing its speed: for the same operation time, it is better to acquire fewer spectra, but to ensure that each of their points is measured several times, thus obtaining more accurate values, in order to avoid uncertainties propagating through the calculation chain with the resulting divergent nonlinear effects.

Although in the best case, the average between actual and retrieved adulterant concentration was 1.7%, which is reasonable with an average standard deviation of the measurements of 3.7%, in a single measurement, the absolute difference between actual and retrieved adulterant concentration reached 14%. It is therefore considered that, at the present stage of development, the technique described in this paper cannot detect less than 15% with certainty, suggesting that the limit of detection should be cautiously set at 20%.

The results for oregano are worse than for saffron (limit of detection of the adulterant in the order of 2%) simply because–despite the improvement of the experimental system and the refinement of chemometric analysis–the spectra of pure oregano and pure olive leaves are similar (they have comparable plant matrices), whereas the spectra of saffron, turmeric and tartrazine have different absorption peaks from each other. However, this limitation is not so serious from a practical point of view because adulteration of oregano of less than 20% is not economically attractive. The measurements presented here for some commercial samples indicate values of olive leaves in the range from 1/3 to 2/3 of the total mass. A different case is saffron, which–if of high quality–can be more expensive than gold.

After all, preliminary results that attempt to retrieve the concentration of adulterant from only two wavelengths are not dramatically worse than the PLS models. Therefore, we are developing an even more compact and faster LPAS system that measures a well-defined adulterant from only two wavelengths. Clearly, the system that acquires the whole spectrum remains superior in terms of flexibility, being able to adapt to the detection of different frauds.

## 5. Conclusions

The LPAS system developed at ENEA to detect food fraud and initially tested with saffron was challenged with a more elusive adulteration obtained by mixing olive leaves with oregano. The first results with standard samples of one olive cultivar and one oregano variety were sufficiently encouraging, even though they showed that the spectra of olive and oregano leaves are morphologically similar.

Representative samples of olive cultivars and oregano varieties widely grown in Italy with economically advantageous adulterant concentrations were then prepared. The spectral measurements of these samples, obtained in a few minutes with laser sensing, were processed with chemometric analysis by repeating the operation twice, either by slightly modifying the samples or by somewhat varying the data acquisition strategy.

In summary, the limit of detection of the LPAS system in its current state of development is better than 20% for a single measurement (obtained by acquiring a spectrum) and in the order of a few per cent for repeated measurements (obtained by acquiring a dozen spectra).

In the future, in addition to the development of a fast and compact system with only two wavelengths, we anticipate further improvements in the experimental system and data processing. E.g., we are carrying out the simulation of the cell with the finite element method, the insertion of a divergent lens before the cell to more evenly distribute the laser beam over the sample, the optimisation of the microphone position and the synchronisation of the chopper with the lock-in amplifier. In addition, we are liaising with top European experts in chemometric analysis within the framework of COST Action CA19145 ‘SensorFINT–European network for assuring food integrity using non-destructive spectral sensors’. Finally, we intend to broaden the scope of the LPAS system, exploring the possibility of detecting chemical compounds such as, for example, nerve agents, of great interest in the field of security.

## Figures and Tables

**Table 1:** Main elements of the LPAS system.

Element	Manufacturer	Model
BS	Thorlabs	WG71050
C	ENEA ^1^	N.A.
CH	Thorlabs	MC2000B-EC
F	Hewlett-Packard	5489A
LIA	Zurich Instruments	MFLI
M	Thorlabs	PF10-03-M02
MP	Knowles	EK23024000
PC	AAEON	ACP-1106
PM	Gentec-EO	UP12E-10S-H5-INT
QCL	DRS Daylight Solutions	MIRcat-1200
W	Thorlabs	WG71050-E4

^1^ The cell has been manufactured at ENEA.

**Table 2:** Main specifications of the QCL.

Wavelength range	6.0–11.1 µm
Linewidth	100 MHz
Wavelength accuracy	1 cm^−1^
Average power	60 mW
Power stability	3%
Spatial mode	TEM_00_
Beam divergence	4 mrad
Beam pointing stability	2 mrad
Spot size	2.5 mm
Polarisation	Vertical 100:1

**Table 3:** PLS results (February 2021).

Actual OL [%]	Predicted OL [%] (Average ± Standard Deviation)	Absolute Difference
0	3 ± 4	3.3
20	19 ± 3	0.7
60	60 ± 4	0.2
80	82 ± 4	1.5
90	91 ± 3	0.8
95	94 ± 4	1.0
100	96 ± 3	4.0

**Table 4:** PLS results (June 2021).

Actual OL [%]	Predicted OL [%] (Average ± Standard Deviation)	Absolute Difference
0	7 ± 7	6.8
20	29 ± 7	9.0
60	52 ± 7	8.5
80	86 ± 7	5.5
90	86 ± 9	4.5
95	94 ± 8	1.0
100	93 ± 8	7.2

## Data Availability

The data presented in this study are available on request from the corresponding author.
